# What John B. Watson Meant to B. F. Skinner: The 1979 Symposium

**DOI:** 10.1007/s40614-026-00498-0

**Published:** 2026-05-29

**Authors:** Bruno Angelo Strapasson, Edward K. Morris

**Affiliations:** 1 Department of Psychology, Federal University of Paraná, Curitiba, Paraná, Brazil; 2 University of Kansas, Lawrence, KS USA

**Keywords:** John B. Watson, B. F. Skinner, History of behaviorism, History of behavior analysis, Radical behaviorism

## Abstract

This article introduces a 1979 symposium held at Furman University in honor of the centennial of John B. Watson’s birth and reproduces B. F. Skinner’s unpublished contribution to it. Watson was commemorated in presentations by James V. McConnell, Fred S. Keller, and Skinner. The introduction describes the symposium’s structure, the presenters’ professional records, and their views on Watson’s behaviorism, situating the speech within its historical context. McConnell offered a critical yet admiring view of Watson, whereas Keller emphasized Watson’s influence on behaviorism’s application, particularly in education. Skinner’s presentation, titled “What John B. Watson Meant to Me,” reflected on both his intellectual debt to Watson and their philosophical differences. This is likely the lengthiest and most in-depth of Skinner’s appreciations and analysis of Watson’s work. In publishing the presentation, the article contributes to the historiography of behavior analysis and clarifies the relationship between two of the most influential figures in the history of behaviorism.

On April 5–6, 1979, Charles A. Brewer, professor of psychology at Furman University in Greenville, South Carolina, convened a symposium to honor the 100^th^ anniversary of John B. Watson’s (1878–1958) birth. Watson was Furman’s most prominent alumnus. He graduated with a Master of Arts in Philosophy in 1899 and received an honorary degree of *Legum Doctor* (Doctor of Laws) in 1919. In the first quarter of the 20^th^ century, he played a central role in the formulation of behaviorism (see Watson, 1913a, b, 1914; see Buckley, 1989; Todd & Morris, 1994). For the symposium, Brewer invited James V. McConnell, Fred S. Keller, and B. F. Skinner as speakers.

## James V. McConnell (1925–1990)

McConnell was the first speaker. He spoke on the evening of the symposium’s first day. He was a professor of psychology at University of Michigan. Although not known as a behaviorist, McConnell was famous for his research on the alleged biological transfer of memory in planaria (e.g., McConnell, 1962). He was also known as the founder of the *Worm Runner’s Digest* (1959–1979), which began as a satirical science magazine and later published actual research. To better accommodate the research, McConnell distinguished the *Worm Runner’s Digest*, the satirical magazine, from the *Journal of Biological Psychology*, an academic journal in 1964. Nevertheless, both periodicals were published together, one in the back of the other. The uniqueness and controversial nature of the journals garnered McConnell considerable visibility (Stern, 2013).

Nonetheless, McConnell referred to himself as a “semi-radical” behaviorist (McConnell, 1985, p. 683), viewing Watson as the truly radical^1^ behaviorist. He described his work as “a result of Watson’s influence on physiological psychology” (McConnell, 1985, p. 690), and wrote a popular textbook, *Understanding Human Behavior* (McConnell, 1974). The textbook was sympathetic to behaviorism, but also historically controversial. For example, it suggested that Watson’s dismissal from John Hopkins University was related to a questionable accusation of improper sex research he had conducted with some students. This was unfounded (see Benjamin et al., 2007). Nonetheless, his book led McConnell to receive the American Psychological Foundation’s 1977 award for Distinguished Contributions to Education in Psychology. Indeed, he was well known for his interest in the teaching of psychology. This was Brewer’s field of expertise, which may explain, in part, why McConnell was invited to speak at the symposium.

In his presentation, “John B. Watson: The Man and the Myth,” he described his relation to Watson (e.g., an admirer, but critical) and what he thought were Watson’s main contributions to psychology: his focus on objective measurement, the importance of the environment, his attention to the physiology, his materialism, and his appeal for psychology to be an applied science. McConnell acknowledged Keller and Skinner on their contributions to psychology, but criticized Skinner’s research and theory, suggesting for instance that Skinner’s behaviorism was mechanistic, which it was not (Morris, 1993). Skinner addressed McConnell’s criticisms in his own presentation. A revised version of McConnell’s presentation was published in *Psychological Reports* (McConnell, 1985).

## Fred S. Keller (1899–1996)

Fred S. Keller was the second speaker. He spoke on the afternoon of the second day of the symposium. Keller was a well-known behavior analyst and Skinner’s friend and academic peer since graduating from Harvard in the late 1920s. He received his doctorate in 1931. Keller’s contributions were numerous (see Keller, 2009). In the 1930s, he wrote a history of psychology (Keller, 1937). During World War II, he devised improved methods for teaching Morse Code (e.g., Keller, 1943). With William N. (“Nat”) Schoenfeld (1915–1996) at Columbia University, he established the first undergraduate psychology course based on operant principles in 1946 (Keller & Schoenfeld, 1949) and co-authored the field’s first textbook (Keller & Schoenfeld, 1950). In the 1950s, he published a book on reinforcement theory (Keller, 1954/1969). With colleagues in Brazil in the 1960s, he developed a notable application of behavior analysis to higher education—the Personalized System of Instruction (PSI; Keller, 1967, 1968). He retired from Georgetown University in 1967 and, in 1977, received American Psychological Association Distinguished Scientific Award for the Applications of Psychology.

Keller mentioned Watson frequently in his writings (e.g., Keller, 1927, 1982, 1983, 1992, 1994, 1996, 2009; Keller & Schoenfeld, 1950). Indeed, his reading of one of Watson’s books, *Psychology from the Standpoint of a Behaviorist* (Watson, 1919), introduced him to behaviorism (Keller, 1994, 2009). In addition, Keller described behavior analysis as a successor to Watson’s behaviorism, albeit with modifications (Keller, 1983).

His presentation at Furman was titled “Applied Behaviorism Since John B. Watson.” In it, he addressed personal similarities between himself and Watson (e.g., both were born in small towns, were raised in the South, had poor academic records as boys, did not like college, and served in the Army during World War I). He promoted aspects of Watson’s behaviorism, for instance, the use of objective methods, an emphasis on learning, the role of the conditioned reflex, and the belief on the importance of the environment in shaping human behavior. In addition, he described developments in the application of behavior analysis, both Watsonian and operant, with an emphasis on educational technologies, for instance, Wolpe’s behavioral therapy, Keller’s code-voice method for teaching morse code, Skinner’s pigeon-guided missiles, training animals to perform in television programs, interventions with psychotic patients, teaching machines, and PSI. His presentation was published in Portuguese in 1983 (Keller, 1983).

## B. F. Skinner (1904−1990)

**Fig. 1:**
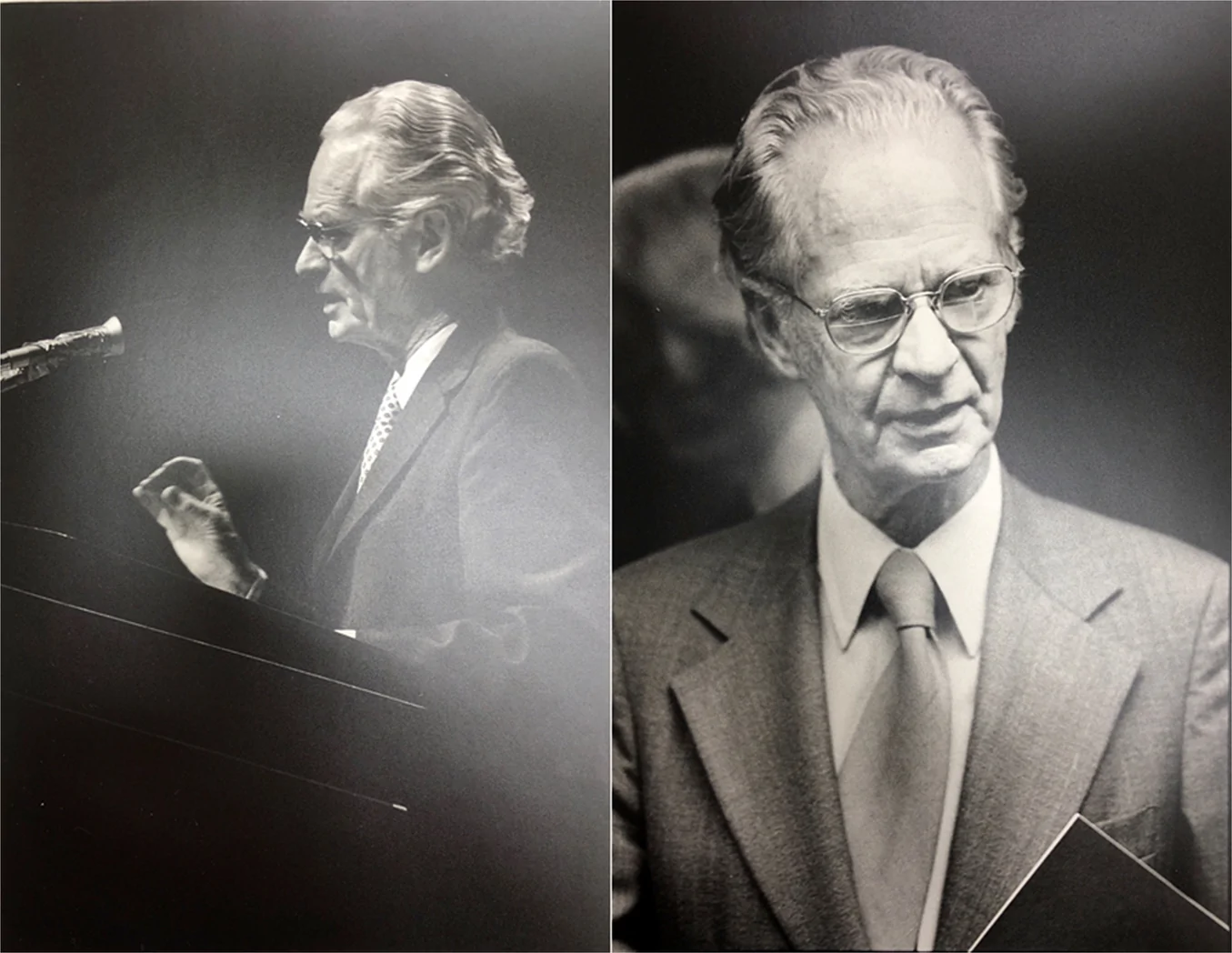
Pictures of B. F. Skinner taken during the 1979 Symposium on John B. Watson sent by Lib Manney, the secretary of the Department of Psychology at Furman University, to Cedric A. Larson. *Note.* Available at Folder 2 “Watson centennial”, Box M1296, Larson (Cedric) Paper, Archives of the History of American Psychology, University of Akron

B. F. Skinner was the third speaker. Pictures taken of him during his speech are shown in Figure 1. He spoke during the evening of the second day of the symposium. In 1931, he received his doctorate in psychology from Harvard University and soon established the field’s basic science—the experimental analysis of behavior (see Skinner, 1938). In the 1940s, he formulated the philosophy of that science—radical behaviorism (Skinner, 1945). In the 1950s, he extended his science and philosophy to interpretations of everyday human behavior (e.g., Skinner, 1953), in particular, verbal behavior (see Skinner, 1957). Throughout, he advanced their application (Morris et al., 2005). He retired from Harvard in 1974, after which he was a professor emeritus. In 1979, he was senior among behavior analysts (see Skinner, 1979, 1983), which likely accounted for Brewer’s invitation.

Skinner’s work was related to Watson’s in many ways (see Moore, 2017). In his autobiography, he mentioned Watson’s (1930) *Behaviorism* as “the book which brought me into psychology” (Skinner, 1983, p. 190), adding “I had, of course, began as a disciple of Watson” (p. 191). Skinner took Watson seriously. In *The Behavior of Organisms* (Skinner, 1938), he wrote that the science of behavior was born when Watson extended Lloyd Morgan’s (1852-1936) canon—the law of parsimony—from animal behavior to the human behavior. Later, he wrote that “It was John B. Watson who made the first clear, if rather noisy, proposal that psychology be regarded simply as a science of behavior” (Skinner, 1963, p. 952). In an obituary published in *Science*, he wrote that Watson’s “brilliant glimpse of the need for, and the nature and implications of, a science of behavior was all but forgotten. Perhaps history is ready to return a more accurate appraisal” (Skinner, 1959, p. 198). However, he followed Watson in only a broad sense.

Skinner also criticized Watson’s behaviorism. When Watson (1930) defined the primary goal of his science as “to be able, given the stimulus, to predict the response or, seeing the reaction take place to state what the stimulus is that has called out the reaction” (p. 16), Skinner (1938) found this “an impracticable program” (p. 10), which he called “the botanizing of reflexes” (p. 10). In its place, Skinner (1931, 1935) analyzed behavior as a correlation of classes (not instances) of responses and stimuli. He also rejected Watson’s definitions of stimuli and responses (Skinner, 1938, pp. 42, 375; 1979, p. 203), theory of language (Skinner, 1957, pp. 87–88; 1968, p. 124; 1979, p. 335), unnecessary denial of introspection (Skinner, 1974), his frequency theory of behavior (Skinner, 1959), and excessive confidence that conditioned reflexes explained complex behavior (Skinner, 1959, 1974). Moreover, Skinner suggested that Watson’s rhetoric about behaviorism attracted the negative attention he tried to counter in *About Behaviorism* (Skinner, 1974; see also Skinner, 1983, p. 267). In that book, Skinner wrote that “the scientific analysis of behavior has made dramatic progress, and the shortcomings in Watson’s account are now, I believe, chiefly of historical interest” (p. 5). Indeed, he did not want to call his science and philosophy *behaviorism* because it was “too closely tied to John B. Watson” (Skinner, 1979, p. 331). These criticisms and the differences notwithstanding, Watson was a significant influence on Skinner, which he described extensively in Brewer’s symposium and titled “What John B. Watson Meant To Me.” In Skinner’s (1983) only mention of the symposium, he wrote:

John B. Watson had attended Furman College in Greenville, South Carolina, and in April 1979 the college (now a university) opened the John B. Watson laboratory. Watson’s son attended the ceremony and Fred Keller and I were among the speakers. Half a century had passed since Fred and I defended Watson against the psychology of Titchener and Boring, and a great deal had happened to support our position. The current science was not exactly the science Watson had projected, but his central theme, “study behavior not mind,” had survived. (p. 380)

The recording of Skinner’s presentation was located during archival research at Furman University conducted for a separate study on John B. Watson. Unlike McConnell’s and Keller’s presentations, Skinner’s has not been published until now. In transcribing and publishing it, the relations between two of the most preeminent figures in the history of behaviorism are further developed and clarified.^2^

## Introduction to the Skinner Lecture

### Charles Brewer

Ladies and gentlemen, welcome to the final lecture of the John B. Watson symposium. With these special events, Furman University honors one of its most distinguished and influential *alumni*. We are pleased that you have joined us for this commemoration. We are delighted that several of Dr. Watson’s relatives are in the audience and that so many Furman psychology graduates and visiting psychologists are also here. At this time, I should like to recognize Dr. John B. Watson’s son, Mr. James B. Watson, who now lives in California. Mr. Watson will you please stand to receive our warmest welcome.

Our speaker this evening received the Gold Medal Award from the American Psychological Foundation in 1971^3^ for his distinguished record of scientific and scholarly accomplishments. The citation read in part:

When historians decide a hundred years from now which psychologists are most deserving of remembrance, it may well be that Burrhus Frederic Skinner’s name will lead the list. An experimentalist whose studies have stimulated countless others, a technologist whose reach has encircled the globe, and a systematist without modern peer. This man’s contributions will not be easily forgotten.

His address this evening is appropriately entitled, “What John B. Watson Meant to Me.” It is my distinct privilege and high honor to introduce to you at this time Professor B. F. Skinner.

## What John B. Watson Meant to Me

### B. F. Skinner

Thank you very much Professor Brewer, ladies, and gentlemen. I did not write a speech to read to you tonight. I knew that if I had done so, half of what was in it would have been anticipated by the other two speakers, so I simply made some notes, half of which I have now crossed out, and the rest of which will, I trust, serve me for my remarks this evening.

I went to Hamilton College in New York State in the early 20s and psychologists there never mentioned Watson. For the best of reasons, there were no psychologists there. Hamilton College had not yet reached that stage of erudition. There was a professor of philosophy who had studied in Leipzig and I think had some contact with Wundt, but the only psychology he taught us was to pull out of the drawer a pair of dividers and demonstrate the two-point limen, hastily returning the dividers to the drawer. That was my exposure to psychology as an undergraduate.

I majored in English and, at Hamilton, that meant the works. I read *Beowulf*^4^ in Anglo-Saxon, Chaucer in the original, a full year of Shakespeare, a year of Restoration drama, and so on. My only real contact with psychology was what I read when I was preparing a term paper on Hamlet’s antic disposition. I tracked Shakespeare back to the original Danish historian, Saxo Grammaticus,^5^ who had a perfectly good reason for having his character, Amleth, feign madness because in those days psychotics were regarded as *Les Enfants de Dieu*, the children of God and, you know, no one dared touch them. So, when Amleth was afraid that his uncle would kill him, he pretended to be mad. That theme disappears in the Shakespeare version, but I analyzed it in great depth and read something and picked up the term *involution melancholia*, which was not relevant at all, but I didn’t know that and I wrote a very good paper on the subject and got an A.

The year after I graduated, I tried to be a writer. I had practiced writing in college and I thought it was my career and my family allowed me take a year off to try my hand. Well, I discovered fairly soon that, although I indeed could write, I had nothing to say. I was enabled to remedy that defect, so I like to believe, by an accident and the accident had to do with a literary magazine that I subscribed to during those college years and the first year afterward—the year I refer to as the Dark Year—a magazine called *The Dial*. It was a marvelous magazine and there has been nothing like it ever since. It was edited and supported by Schofield Thayer, edited by Marianne Moore. Every issue had in it a colored picture tinted in coloring. Colored reproduction was very bad in those days, but there was a picture and the remarkable thing was that they [sic] were modern. They were the kind of thing that one almost never saw. There were Impressionist and post-Impressionist. This one^6^ is by Pierre Bonnard—*Le Jardin*. The magazine had a roster of famous writers in this issue. There’s a story by D. H. Lawrence, very good articles by names that are now not so well known, but were very prominent in those days: Roger Fry, Alyse Gregory, Whitter Binner, Llewelyn Powys, Robert Morris Lovett, and so on.

But in this issue, which came out in August 1926—I was 2 or 3 months embarked upon this disastrous year trying my hand as a writer—in this issue, there is a review of Ogden and Richards’s *The Meaning of Meaning.*^7^ Now, that was a book I had heard about because, although I wanted to be a writer of fiction, I took writing as a craft seriously and studied it rather carefully—and I had heard this book discussed. This review was written by Bertrand Russell.^8^ Now, Russell was, of course, already a distinguished figure. He and Whitehead had written the famous *Principia Mathematica*^9^ and then Russell had gone his own way with prominent political and other ethical and moral affairs. He’d written a book, *Marriage and Morals.*^10^ During the First World War, he had protested the war and been put into prison—and he continued. When he was in his 90s, he continued to protest, in that case it was Vietnam and the atomic bomb, and he would go, an old man in his 90s, to sit in Trafalgar Square having put an air cushion in his trousers before going there.

The review took the book quite seriously. It said this is an important book for three reasons: It’s an important subject; the treatment is scientific rather than mythical; and some of it may be right. Then, he set down his own ideas about language or verbal behavior and he made the following eight points: Words are social, in other words, you talk to people; words are bodily movements; words are means of producing effects on others; words are caused by stimuli; heard words are stimuli; it is not of the essence of language to express ideas; the distinction between logical and emotive use is illusory; and, in the individual, a child has a heard language before a spoken [sic].

At the end, in a footnote, Russell said “It will be seen that the above remarks are strongly influenced by Dr. Watson, whose latest book, *Behaviorism,*^11^ I consider massively impressive.” Well, that was high praise from an extremely important person at the time and, naturally, I began to ask: Who is this man, Dr. Watson, who was not otherwise indicated or identified in the article? I had to find out the rest of his name elsewhere. Well, I also bought Russell's book, *Philosophy*, that was called in England, *The Story of Philosophy,*^12^ but Will Durant^13^ had usurped that title in America, so it’s called here just *Philosophy*. It is not an important book and students of Russell pay little attention to it. Russell's biographer says that it was dashed off at speed for the American market.^14^ But it begins with seven chapters which, are really quite extraordinary.

The first one is simply called “Man in his Environment.” That sounds so commonplace today; it’s hard to realize quite what it meant in those days. But this was Watson, this was human behavior in response to the environment. The second one: “The Process of Learning in Animals and Infants.” There again, you have a bridging of the lower animals with humans. And, there’s a chapter on language, in which he develops the ideas he had developed in the review. Then, a chapter called “Perception Objectively Regarded”; another one, “Memory Objectively Regarded”; and an extraordinary one, “Inference as a Habit.” Here was a man who had written the great work on logic—*Principia Mathematica* along with Whitehead—and now he was talking about inference as a habit. And finally, a chapter on “Knowledge Behavioristically Considered.” Well, this was really something, and I was turning away from my failure as a writer in the direction of something which interested me.

This was a very sophisticated epistemological analysis of the heart of behaviorism, much more sophisticated than Watson. Although Watson himself had some philosophical training, and was certainly no tyro, but Russell was older and prestigious, and these chapters struck me as extremely important. Many years later, when I met Lord Russell, I told him it was his book that converted me to behaviorism. “Good heavens,” he said, “I thought that book had demolished behaviorism.” Well, the reason that it had not demolished my faith in behaviorism was that I never finished the book. The chapters that I have just given you the titles of were in the first part of the book. The middle part was a rehash of Russell’s views of the nature of the physical world, and the last half was a mentalistic attack on the first part, in which Russell thought he had reinstated the mentalism in some way. But, when I got to that middle part, I recognized it as old stuff. It really was cooked up for the American market, I’m sure, and so I stopped reading it and it left me in the position of a behaviorist.

And, of course, I turned directly to Watson and bought his book *Behaviorism*, and that, of course, made an enormous difference and I became an instant behaviorist, so much so that before the year was out, when an endocrinologist named Louis Berman wrote an attack on *Behaviorism*—called “The Religion Called Behaviorism.”^15^ Berman was an endocrinologist who had written a book called *The Glands Regulating Personality*.^16^ I took up the cudgels immediately and wrote a review. I knew nothing about any of this, of course, but I was a charter subscriber to the *Saturday Review of Literature* and I wrote an attack on Berman defending Watson and sent it off. I never heard from the *Saturday Review* about it.

Then, I realized that my future as a writer of fiction was ended and that I should be turning to psychology, and I applied to Harvard and was admitted. I wouldn’t be admitted today, but conditions were more lenient at that time. But I had some time left before going and so I went to Greenwich Village where I sowed a few wild oats and worked in the bookshop. And, on the shelves in the bookshop was Watson’s *Psychological Care of Infant and Child*^17^ and I read that between customers, holding it in such a way that I wouldn't dogear it or cause it to be unsellable.

Well, then I arrived at Harvard having read Russell, *Behaviorism*, and *The Psychological Care of Infant and Child*, and I regarded myself as a full-fledged behaviorist, but I knew of course very little about it. However, in that first year, I took a seminar with Walter Hunter; as Professor Keller has mentioned. Hunter came in from Clark University once a week and conducted a seminar talking about behaviors which seemed to indicate symbolic processes. It was the thing that Köhler had talked about in *The Mentality of Apes*,^18^ but Hunter used American works—Yerkes among others—and it was a good exposure to hard-headed behaviorism [sic], because Hunter was one of three people who wrote articles during the 20s which were classical in the statement of the behavioristic position. Lashley, Tolman, and Hunter wrote excellent articles on various aspects of behaviorism.

I also knew two graduate students who were behaviorists. One of them, Charles K. Trueblood, who had written book reviews for *The Dial*, and I used to admire that name across the head of the page—“Charles K. Trueblood.” It looked very regal and now I found, in carrying cages of rats and working on the rotation of the maze, it was reassuring. The other graduate student was, of course, Fred Keller. And I remember those early colloquia when I, as I reported to my parents, kept the place in an uproar for an hour and a half after I gave a paper. And bear in mind I only had two halves of courses in psychology at the time. But that didn’t keep me from defending a behavioristic position and most of the time that Fred Keller would come to my rescue in those early years and save me as I floundered. But the questions that we debated at the time were such things as “Where is red?,” “Is it out in the world?,” “Is it in the sensation?,” and so on. And I never had the answers to those technical questions and, fortunately, Fred Keller had.

And when I began to do experimental work, I didn’t really get very much help from Watson. As two speakers have made clear, Watson wanted a science of behavior, but that’s not enough. You can’t simply conjure one up. You have to do something and get results, and Watson had very few results to offer at that time. He picked up Pavlov’s conditioned reflex and made a great deal of that, but for the rest, he stayed with the concept of habit, which is not a very profitable concept. He believed that we tended to do those things we’ve most often done. When a rat goes through a maze successfully, you know that it has made all the right turns. It may not have made all the wrong ones, so that in the long run, as it goes through again and again, it must have made the right turns more often than any other and this lays down on a kind of path which is a habit and that is why the rat turns and is able to run the maze. That was not an appealing notion to me and, moreover, I quickly came under the influence of physiologists.

There was a strong Department of General Physiology at Harvard, the head^19^ of which tended to proselytize and recruit young people from psychology over the objections of the Department of Psychology, and I got in on that. I studied Sherrington; Magnus, who worked on postural local motor reflexes; and of course Pavlov. I had picked up a copy of Pavlov^20^ before coming to Harvard. Actually, I read it during those months in Greenwich Village, where I paired it with various exciting stimuli of one sort or another, and that is why the book still arouses many strange reactions in me, as Pavlov would have predicted, of course.

But, I began in what was an attempt to analyze behavior from the point of view of the reflex with a little simple device. First of all, I worked on how rapidly a rat eats the pellet of food repeatedly during a period in which it eats its fill. And the rate at which it eats, drops slowly as the rat eats. And I wanted to get some little response, so I bent a wire and made a lever and that was the beginning of the so-called lever box. And I went on from that to do a kind of research which was not at all what Watson had been talking about or Pavlov for that matter. And since I don't believe that Professor McConnell got this quite straight last night, I want to take a little time to try to say exactly what I feel that early operant work represents.

We’re often accused of treating animals as machines and that is, no doubt, what early reflex physiologists were doing—what stimulus-response psychologists are doing. It was a “push the button, get behavior” kind of thing. But viewing an animal as a machine is something that an operant analyst cannot possibly do because he or she appeals to a principle which is found only in living things and that is the principle of selection by consequences. It began with life itself when those first molecules fell together in that helix or whatever it was in those days and, when that large molecule suddenly was able to reproduce itself from the material about it, then what we call life began. And with reproduction, there became possible another kind of selection because, if other entities are produced through reproduction differ, some of them will be more likely to have the capacity to reproduce under a greater variety of circumstances and you end with the evolution of millions of species. Natural selection is the first great example of selection by consequences and part of what is selected is, of course, behavior. If you watch those excellent animal films which are now being shown, at least in the Boston area, on public television—“Wild, Wild World of Animals”^21^—you will see fantastic examples of elaborate instinctive behaviors and there’s no question what is meant by instinct there. These are the behaviors which have evolved. Just as respiration, digestion, circulation evolved, so do behaviors with respect to the environment. But that kind of behavior only works if the environment is reasonably stable from generation to generation, and that doesn't allow behavior to get very far.

What then happened was the evolution of operant conditioning which is a process, in the individual, in which behavior is selected by the consequences in the life of the individual, rather than by survival as a consequence in the evolution of species. So that you now have another kind of selection that is not feedback. Reinforcement is not correctly represented with the word “feedback.” Nothing feeds back in the natural selection. The organisms that are bred and produce offspring are out of the picture. Nothing goes back to them. What is followed will or will not survive and reproduce its kind. And the same thing is true in operant behavior: The response which is reinforced is now a matter of history and has not changed. What has changed is the probability that future responses will occur.

That is a principle which Watson was not able to take advantage of because he had not thought it through or had not seen the kinds of experiments that make the principle absolutely inevitable as an item in a scientific collection of laws. I think the difficulty was that Watson was inclined to reject Thorndike and the Law of Effect because Thorndike was not a behaviorist. Thorndike appealed to feelings, satisfactions, and annoyances. Watson was rather harsh in characterizing Thorndike’s theories. At one point, he said: “Thorndike appears to believe that kind fairies build the connections between stimulus and response.”^22^ Well, having given up a very promising principle, the Law of Effect, Watson was left with habit as a product of frequency and, of course, the conditioned reflex, which Mary Cover Jones, Rosalie Rayner, and others lead to, as Professor Keller said this afternoon, many current techniques, in behavior therapy.

I would, necessarily then, diverge from the kind of science which Watson anticipated and, moreover, the concept of a behavioristic philosophy of behavioral science had fallen into disrepute. In an interview in the forties, when someone asked me, “Do you consider yourself a behaviorist?”, I said no, that although I was a student of behavior, I was not actually a behaviorist^23^. And I think this was my unwillingness to use the term because of the bad repute into which it had fallen.

Some of this was the rise of ethology and an attack on the environmentalism of Watson. I will not quote to you the full passage about “Give me a dozen healthy infants,”^24^ but it no doubt caused trouble. Although Watson said immediately that he was exaggerating, it was taken to be based on faith in the environment and a denial of a genetic contribution. If you look at it closely, what he was denying was not a genetic contribution, but traits of one kind or another, the ideas, traits of character, and so on said to be part of human nature and responsible for behavior. He was simply taking the line that, as Professor Keller pointed out, that a great deal can be done with an organism regardless of its genetic endowment. And, of course, Watson, himself, as Professor McConnell pointed out, was an early ethologist, whose work with noddy and sooty terns certainly ranks as among the very first work of that kind in ethology.^25^ But at that time I’m talking about, in the forties, behaviorism was really not widely accepted. Also, of course, Dr. Spock had come along with a new method of child care and Watson's principles in the *Psychological Care of Infant and Child* were beginning to seem very hard [sic], distant, and uncaring. I’m very glad that Professor Keller pointed out that that was not true of Watson himself. And, as I think Professor McConnell said with certain timidity that “he made a difference” or it may have been Mr. Watson himself who pointed it out.

Anyway, in the 40s, Watson was not a popular figure and I had abandoned my crusading spirit partly because my own scientific work was not very close to Watson’s own theories about behavior. But that changed and, as I began to be aware of the practical applications of an operant analysis, I began to go back to Watson again because he was, of course, extremely optimistic about what could be done in the field of human affairs. And by the time that I wrote his obituary for *Science Magazine*^26^ in the late 50s, I had really again begun to call myself a behaviorist and I wrote a paper “Behaviorism at fifty” in 1962,^27^ which celebrated Watson’s original proposal, as a suggestion that we create a science of behavior. And a few years later, in the paper on the phylogeny and ontogeny of behavior,^28^ I defended Watson’s position in ethology and the importance of the environment.

By 1974, I had written a book *About Behaviorism*,^29^ which, although it doesn’t mention Watson in many points, was an effort to put into good form what I thought the current philosophy of a science of behavior should be. I did make it quite explicit in the very first sentence of that book: “Behaviorism is not the science of behavior, but the philosophy of that science.” I think that is the right way to put it and it leaves Watson in the right place because Watson, like Russell, was not giving you the details—the laws and the concepts of a science. They were talking about the possibility of the science and its extraordinary implications. Watson saw the need to treat behavior as such, rather than as the expression of something else, such as a mental life. And if what you do is simply what you do because you have a purpose or an intention or any of the mental entities that you suppose an active will, if that is what behavior is, then of course it is not a subject matter. But if you turn to behavior in a biological sense—simply is what an organism is doing—and then ask whether these old concepts are worth considering, you are engaging in a philosophical analysis rather than scientific one. Of course, what you say is based upon the facts of a science of behavior, but the argument for behaviorism is really a defense of the science and a statement of its implications. It was the only thing Watson could do because he could not create the science instantly. It is what we do now when we use what we have learned in the laboratory to interpret behavior as we observe it elsewhere and that notion of interpretation is very important.

I disagree with McConnell again when he says that the essence of behaviorism is to concern oneself only with those things which can be measured. I don’t know whether Watson actually said that. The people who did say it were the logical positivists who, in the early 30s, beginning in the Vienna Circle^30^ and, emigrating to this country, they continued to say that. Carnap, Tarski, my friend Herbert Feigl, and others, they believed that only those events which could be observed by two people can possibly be part of science. Hence you rule out those events which the individual may observe in himself. They must be left out and that is the argument of methodological behaviorism: that there is such a thing is mental life, but we can’t deal with it. I, as a radical behaviorist, am interested in examining the extent to which what an individual sees about his own body, feels his own body to be, is important to consider. I can’t deal with it with complete certainty, but to leave it out is a great mistake.

In 1945 in a paper called “The Operational Analysis of Psychological Terms,”^31^ I examined the ways in which a verbal community could teach an individual to talk about private events going on in his own body—events which, obviously, the community cannot see. There are ways in which you can teach a person to distinguish between states of his own body. It’s much more difficult than to teach a person, for example, to distinguish between two colors because those who are teaching also know about the colors. But, there still are ways in which a fairly reliable vocabulary describing privacy, describing private events, can be built up. But that doesn’t mean that you’re describing sensations or perceptions of anything mental. What you are talking about are states of your own body.

And this is an issue that used to come up in those colloquia because someone said once that Watson had no visual imagery. I don’t know whether that is an authentic report or not^32^, but I would agree that he hasn’t, [sic] nor have I, nor have any of you. That is I think the most difficult behavioristic principle to expound. The Ogden and Richards’s book, as Russell pointed out in his review, says things like this: “We may lay it down generally that, whenever we use a word, . . . there is some sensation or image . . . which has frequently occurred at the same time as the word and now, through habit, causes the word.”^33^ That is the kind of thing that was said quite regularly and widely in those days. But how do sensations cause words and so on. There are all sorts of problems there. Watson had visual imagery in the sense that he could close his eyes and say, “I see something.” We all can do that and I’m sure Watson could too, but that isn’t the issue. The question is whether we’re seeing an image. Even when you have your eyes open are you seeing an image. That’s a very difficult question to make clear, but I think the answer is perfectly clear.

Plato, who didn’t understand how you could see something across a room, assumed that you had to be seeing a copy in the back of the head because you had to be intimate with what you knew. “To know” meant “intimacy” in the biblical sense of knowing a woman, extremely intimate, and you had to have things somehow or other in your possession to know them. So, the Greeks created this elaborate fiction that we have, inside ourselves, copies of the world around. Now I’m not the only person who objects to copy theory. Many philosophers do. If you assume that when you see, as you see me here, you were seeing me and not a picture of me inside your head somewhere, and if you close your eyes and, not too well, but well enough, still see me it is not that we're then seeing a copy, but that you are doing with your eyes closed more or less what you were doing with your eyes open, although not as well. And that is, I think, all there is to it. If something has to go on beyond a picture, it can be out in the open, it can be in the back of your head somewhere, but it still has to be seen, and that is not making a copy of it and I believe it’s perfectly correct to say that there are no images in our heads, although it is quite correct to say that I can see things with my eyes closed. With a little practice you can do pretty well.

The story of the way in which in Whistler^34^ taught his students to the paint is relevant. He put the model in the basement and the students had their canvases on the first floor. They would go down and look at the model and come up and painted there. Then he moved them up the second floor, and the third floor, and finally the fifth floor. Well, then they looked very carefully indeed to avoid running up and down stairs and would carry, as we would say, an image. What they merely did was to be able to see the model when they were on the fifth floor while all of the models were in the basement. It doesn’t mean that there was an image anywhere, you see, until it gets on the canvas. That is, I think, a perfectly good answer to the question of whether there are images. And I think we can say that Watson didn’t have imagery and nor do we. I’m sure that it is an answer to the kind of question that Fred Keller and I used to debate in those old colloquia.

Another side of the thing is not the way in which we use the science to interpret older philosophies of human conduct, but the way in which we use it to interpret the world around us and understand why people do what they do. This is the sense in which astronomy interprets the radiation coming from space. Not much you can do about it. You certainly can’t control it. There are only a very few things about it you could try to predict and yet the astronomer can say more sensible things about it than anyone else. And they can do that because of laboratory science. The physics of high pressure, high temperatures, radiation, and so on makes it possible to look at these patterns coming from space and talk about them much more usefully, much more consistently, than would ever be possible otherwise. And if you simply don’t bother with the laboratory fact, but go out and look at the stars, you are not going to give a very good account of what is going on. Or even looking at the radiation records that come in, it’s not until you look at the controlled circumstances in the laboratory that you are able to make sense of the uncontrolled outside. And I think that what we have done with the laboratory analysis is to provide a vocabulary, a set of principles, which make it much easier to see what is happening when the student isn’t learning, when people are gambling, when people do this, that, and the other thing. We watch people behave because of schedules of reinforcement, which we can now identify because we’ve studied schedules in the laboratory, even though for thousands of years the notion of scheduling was simply not understood. We can then, I believe, not only develop the techniques of applied behavior analysis that Professor Keller reviewed this afternoon, but we can begin to design a better way of life. And I think it was that kind of thing that brought me back to John Watson because he was dreaming of a better world—a world in which we were acting scientifically rather than mythically, to use Russell’s words—in improving our lot.

I made my first essay in that direction when I wrote *Walden Two*^35^ and I think it is quite relevant that *Walden Two* was written only a year after our old Project Pigeon^36^ failed because in Project Pigeon we did realize the possibility of very precise control of behavior. And my first move was not to go out and become some kind of tyrant, but to design a world in which there was no tyrant, there was no leader. It was simply an environment so designed that it would induce the people living in the community to behave well, with respect to each other, productively, creatively, and so on. And I believe that you can interpret the whole realm of applied behavior analysis in a very similar way. It is, I believe, our chance, our aspirations, to produce a non-punitive society.

It’s fantastic the extent to which we all still resort to punishment. In the classroom, the teacher is much more likely to criticize the mistakes of students than to look for chances to commend them. In daily life and our relation with others, we are always reminded, when we are annoyed, to complain. We are seldom reminded, when we are pleased, to say anything about it, so that we tend to be punitive in our treatment of other people. Governments are defined as the power to punish. Industries appear to be using positive reinforcement when they pay wages, but what actually happens is that a predictable agreed-upon wage simply establishes a way of life from which a supervisor can disconnect you, if you do not behave in a given way. So, people work on Monday not for the paycheck on Friday, but to avoid being fired and they won't receive that paycheck. Almost every major field of human affairs is still largely punitive. It is certainly true of our international affairs. We talk about aid, but the main things are the planes we sell, the fighter planes, and the nuclear stockpile. Now, is there any hope of getting rid of punishment? Why should we continue to engage in practices which cause so much suffering?

Through the development of agriculture, we have eliminated for many people the sufferings of famine. Through the technology of medicine and sanitation, we have greatly reduced the sufferings which are due to illness and disease. Through the physical technology, we have reduced the suffering associated with hard labor, exhausting work. Can we now, with the technology of behavior, reduce that one surviving source of suffering: the ways in which we treat each other? Behavior modification, unfortunately, is determined [sic] and often used, in connection with drugs, the vomit therapy, as in “Clockwork Orange,”^37^ implanted electrodes, and so on. But it was first used, and still I think should be restricted to describe the use of positive reinforcement. And I think we are gradually learning how to find alternatives to punishment.

One of the things about programmed instruction, and that Professor Keller didn’t mention, is the extent to which it maximizes success. By reducing a subject matter into small units, you can get to the point at which the student is almost always right. And he or she will proceed through a program almost undeterred by failure. And such a program induces strong positive feelings of happiness, of satisfaction, of the absence of depression. Depression is usually due to the absence of reinforcing contingencies. When people move to new cities, for example, they can no longer use the repertoires which were successful where they lived before. They can’t call the grocery store with that number; they can’t drive to the supermarket in that direction; theatres are not where they ought to be; restaurants are not there; and so on. A whole set of behaviors are unable to be executed and a whole new set must arise. And it is not going to be successful for some time and it’s very characteristic of people to move from one city to another to suffer depression. And it’s simply a matter of missing reinforcements. And the kinds of anxiety, depression, violence, and rage which come about from punitive contingencies are the products of those contingencies and will never be eliminated until you turn to different contingencies.

Schools create the problems they then try to solve because it is still true, unfortunately, except where personalized instruction is really being used, that students study to avoid the consequences of not studying. The effect of that inside of high schools is vandalism, attacks on teachers, truancy, and all of the other by-products of aversive control. If you can then redesign a school setting so that behavior is frequently reinforced and progress is made—and progress itself being the major reinforcer—the whole thing changes.

I recently visited some schools in California in which they have learning centers that I had a small part in designing some years ago. These are centers which teach reading and they do that no matter what family background or original language. In one school I visited, the center had, at the moment, working into [sic] the class of students who are about equally divided in four ways: Chicanos,^38^ Asians, Blacks, and White Americans. And they were all learning to read because they were going through material properly programmed. They had earphones on; each child was working on the unit for which he or she was ready; there were cassette earphones; and the workbook which had a chemical magic ink response, which told the student immediately whether he or she was right, and the students work through that material. They like it. They are aware of progress they are making. When the school was vandalized, those rooms were never touched. They’re good rooms. They don’t start up any of the counter-violence. They don’t lead to truancy. That’s the kind of language [sic] Professor Keller is arranging at college level and, I’m happy to say, also at high school and grade school level in some cases. Is the kind of thing which can be done to replace essentially punitive methods with positive reinforcement. And it will lead to a reduction in violence. It will lead to the much more effective acquisition of behavior. We saw that there’s no reason at all why everyone in America cannot learn to read easily, quickly, and to a point at which the natural reinforcers of reading take over, namely, the interesting things in the books that are read. Same thing is true, I think, in government, although it’s very hard.

This morning at a press conference I pointed out how the only proposals taken seriously at the moment to reduce the consumption of energy were aversive. You either ration gasoline and punish the black market or you put on very high taxes so that only the rich can drive. These are aversive methods and someone questioned: How would you reinforce positively the behaviors that would also minimize the consumption of energy? Well, I was not really prepared for that question, but one thing I suggested was that if you can make life more interesting in the home, people won’t be driving around the countryside every evening as they now do. And one way to do this would be to build up excellent public television so that there would be really worthwhile things to see if you stay at home. Professor Keller immediately raised the question of whether or not you couldn’t go beyond that and develop skills, and talents, and knowledge, which would be the interesting thing one can do in support of [INAUDIBLE] and so on, or [INAUDIBLE] measures to be taken. This is true of any technology. You may know to build bridges and you may know all about steel and so on, but before you build one bridge, you have to look at the situation: how wide is the river, how firm is the ground on both sides, et cetera. The same is true in solving any problem. It isn’t necessarily true that I could come up with solutions to a question like that, but I think there are solutions which ought to be explored.

And what bothers me is that we still turn to the punitive methods, as if those were the only effective ones that governments should use. It’s very hard to make it clear that when you reduce unemployment, you are attacking the problem of crime. Or you attack it with more police or with an intensification of the punitive side, as in that movie “Scared Straight,”^39^ you are simply going along the old line. But there are ways in which you can reduce crime by reducing the situations which that lead to it and allowing the individual to behave in ways which are reinforced in the same ways which are not illegal. If you can’t get a job to earn the things you want, then you’re inclined to steal them. But can we not keep our eye on the positive possibilities rather than falling back so often on the punitive?

And I think it’s quite clear what Watson was talking about, particularly in the book *Behaviorism*, although he did not see at the time the principles that would make this possible, would be a redesign of the world as a whole, which would lead in the long run to a solution of the problems which are generated by punitive techniques. The world is in a very bad way. Violence almost everywhere and I think that violence is due to the fact that we still are primarily turning to punitive methods. Now, if the application of a behavioral analysis, beginning perhaps in a very small scale, nibbling at the problem here and there, if that leads us slowly to a reduction in the extent to which punishment works and to the substitution of the kinds of controlling principles under which we all feel free and happy, then I think that will represent the proposal which was at the heart of the Watsonian behaviorism. It couldn’t be stated that clearly at the time and I’m not even stating it well now, but I think we are moving definitely toward a less punitive, if not a nonpunitive, world. And it could only come about if we understand behavior better. And, of course, that was Watson’s great revolution: that there is such a thing as a science of behavior and that, once it is been furthered, great things can be done. Thank you very much.

## Closing Remarks

### Charles Brewer

In closing let me express sincere thanks to our three speakers for participating in the activities of this historic and memorable week at Furman University. My hope is that psychology will continue to flourish during the next 100 years and that all our work will reflect credit upon John Broadus Watson, whom we had honored here.

Thank you for coming and goodnight.

## Footnotes

1 Here McConnell used “radical” in the sense of extreme, rather than in Skinner’s sense of the term as referring to a thorough or fundamental analysis of behavior (see Schneider & Morris, 1987).

2 Transcribing presentations is a challenging task. Spoken and written language are typically different in structure, vocabulary, and style. We sought to preserve Skinner’s presentation as he gave it, while eliminating inessential word repetition (e.g., “I, I” or “well, well”), rephrasing certain clauses (e.g., between 00:14:10 and 00:14:12 of the recording Skinner says “I said to him, I told him it was his book” we eliminated “I said to him” and retained only “I told him it was his book”), and making minor adjustments to improve its written form (e.g., between 00:11:30 and 00:11:35 of the recording Skinner says “Well, I bought also Russell's book,” which we changed to “Well, I also bought Russell's book” to facilitate the reading). Where we could, we allowed the original to stand with a [sic]. As noted in the transcription, a 20-s segment (00:57:56–00:58:16) of Skinner’s presentation is missing or garbled, likely due to a problem in the audio recording (e.g., a mechanical failure or a dropped microphone). We were unable to restore it, even with audio enhancement tools such as Audacity and Adobe Audition.

3 Burrhus Frederic Skinner: American Psychological Foundation Gold Medal Award. (1972). *American Psychologist*, 27(1), 71–72. http://dx.doi.org/10.1037/h0020444

4 Fulk, R. D. (Ed. and Trans.) (2010). *The “*Beowulf*” manuscript*. Dumbarton Oaks Medieval Library 3. Harvard University Press.

5 Friis-Jensen, K. (Ed.) (2015). *Saxo Grammaticus (Volume 1)**: **Gesta Danorum: The History of the Danes* (P. Fisher, Trans.). Oxford University Press

6 Skinner apparently displayed a copy of the magazine when making that mention.

7 Ogden, C. K., & Richards, I. A. (1923). *The meaning of meaning: A study of the influence of language upon thought and of the science of symbolism.* Harcourt.

8 The original review was published in the Volume 81 (1926, July–December issue) of *The Dial* (pp. 114–121). See a republication in Wood, W. S. (1986). Bertrand Russell’s review of the meaning of meaning. *Journal of the Experimental Analysis of Behavior, 45*(1), 107–113. https://doi.org/10.1901/jeab.1986.45-107

9 Whitehead, A. N., & Russell, B. (1910–1913). *Principia mathematica* (Vols. 1–3 volumes). Cambridge University Press.

10 Russell, B. (1929). *Marriage and morals*. Liveright.

11 Watson, J. B. (1930). *Behaviorism* (2^nd^ ed.). Norton.

12 Actually, the book was entitled *An Outline of Philosophy* and not the *Story of Philosophy*. Russell, B. (1927). *An outline of philosophy*. George Allen & Unwin.

13 William James Durant (1885–1891) was an American journalist, historian, and philosopher.

14 The reference is probably to Clark’s biography of Russell, mentioned in Skinner’s autobiography (1983, p. 395). See Clark, R. (1975) *The life of Bertrand Russell*. J. Cape. See also Skinner, B. F. (1983). *A matter of consequences*. Alfred A. Knopf.

15 Berman, L. (1927). *The religion called behaviorism*. Boni & Liveright.

16 Berman, L. (1922). *The glands regulating personality*. Macmillan

17 Watson, J. B., & Watson, R. R. (1928). *Psychological care of infant and child*. Norton.

18 Köhler, W. (1927). *The mentality of apes* (Ella Winter, Trans. 2^nd^ ed.). Kegan Paul Trench Trubner and Co., Ltd.

19 The head of department mentioned by Skinner was William John Crozier (1892–1955).

20 In page 4 of Skinner’s second volume of his autobiography (Skinner, 1979) he suggests that this book was Pavlov’s *Conditioned Reflexes* of 1927. Skinner, B. F. (1979). *The shaping of a behaviorist.* Alfred A. Knopf. See also Pavlov, I. P. (1927). *Conditioned reflexes.* Oxford University Press

21 "Wild, Wild World of Animals” was a commercial syndicated television series about wildlife presented between 1973 and 1978. For more information about this kind of series and its relations to the science scene, see LaFollette, M. C. (2013). *Science on television: A history*. University of Chicago Press

22 In *Behaviorism* Watson (1930) said "Only a few psychologists have been interested in the problem [of habit formation]. Most of the psychologists, it is to be regretted, have even failed to see that there is a problem. They believe habit formation is implanted by kind fairies. For example, Thorndike speaks of pleasure stamping in the successful movement and displeasure stamping out the unsuccessful movements” (p. 166).

23 This interview is mentioned also on the second volume of Skinner’s autobiography (Skinner, 1979, p. 331), but no other details of the interview are provided.

24 The full passage can be found at *Behaviorism* (Watson, 1930, p. 82.)

25 About Watson’s early work on comparative psychology see Todd, J. T., & Morris, E. K. (1986). The early research of John B. Watson: Before the behavioral revolution. *The Behavior Analyst, 9*(1), 71–88, and Dewsbury, D. (2013). John B. Watson's early work and comparative psychology. *Mexican Journal of Behavior Analysis, 39*(2), 10–33.

26 Skinner, B. F. (1959). John Broadus Watson, behaviorist. *Science, 129,* 197–198. http://dx.doi.org/10.1126/science.129.3343.197

27 This paper was published the next year: Skinner, B. F. (1963). Behaviorism at fifty. *Science, 140*, 951–958. http://dx.doi.org/10.1126/science.140.3570.951

28 Skinner, B. F. (1966). Phylogeny and ontogeny of behavior. *Science, 153*, 1205–1213. http://dx.doi.org/10.1126/science.153.3741.1205

29 Skinner, B. F. (1974). *About behaviorism*. Alfred A. Knopf

30 To know more about the relation between behaviorism and logical positivism see Smith, L. (1986). *Behaviorism and logical positivism: A reassessment of the alliance*. Stanford University Press

31 Skinner, B. F. (1945). The operational analysis of psychological terms. *Psychological Review*, *52*, 270–277. http://dx.doi.org/10.1037/h0062535

32 In Watson’s Behaviorist Manifesto, he writes in a footnote “closer examination leads me to deny in my own case the presence of imagery” (p. 173). This assertion was revised later by Watson and was widely discussed in academic literature. Watson, J. B. (1913b). Psychology as the behaviorist views it. *Psychological Review, 20*(2), 158–177. https://doi.org/10.1037/h0074428

33 See p. 111 in Wood, W. S. (1986). Bertrand Russell’s review of the meaning of meaning. *Journal of the Experimental Analysis of Behavior, 45*(1), 107–113. https://doi.org/10.1901/jeab.1986.45-107

34 Here Skinner makes reference to James McNeill Whistler (1834–1903), an impressionist American painter.

35 Skinner, B. F. (1948). *Walden two*. Macmillan.

36 See Skinner, B. F. (1960). Pigeon in a pelican. *American Psychologist*, *15*(1), 28–37. http://dx.doi.org/10.1037/h0045345

37 Kubrick, S. (Director & Producer) (1971). *Clockwork Orange* [Film]. Polaris Productions & Hank Films.

38 Even though the word “Chicano” may have a pejorative connotation today, it was used by civil rights movements in the United States during the 1960s and 1970s as a symbol of identity and the fight for rights (see Rodriguez, M. S. (2015) *Rethinking the Chicano Movement*. Routledge).

39 Shapiro, A. (Director & Producer) (1978). *Scared Straight!* [Film] Golden West Television
